# Proximity patterns in water buffaloes' activities on pasture

**DOI:** 10.5194/aab-63-19-2020

**Published:** 2020-01-17

**Authors:** Eleni T. Tsiobani, Maria D. Yiakoulaki, Nikolaos D. Hasanagas, Ioannis E. Antoniou

**Affiliations:** 1 Department of Forestry and Natural Environment, Faculty of Agriculture, Forestry and Natural Environment, Aristotle University of Thessaloniki, 54124 Thessaloniki, Greece; 2 University Forest Administration, Faculty of Agriculture, Forestry and Natural Environment, Aristotle University of Thessaloniki, 54124 Thessaloniki, Greece; 3 Department of Statistics and Operational Research, School of Mathematics, Aristotle University of Thessaloniki, 54124 Thessaloniki, Greece

## Abstract

Water buffaloes are considered social animals and perform several activities on pasture, such as grazing, moving, standing, ruminating, wallowing, lying, and drinking. However, the way these animals form their social structure in the herd during each one of these activities is still unknown. Literature for water buffaloes has focused mainly on their productive characteristics, impact of grazing on wetlands and behavior during grazing but failed to address the way these animals form their social organization during their activities on pasture. In this study, the tools of social network analysis are used to analyze, detect, and depict the proximity patterns in water buffaloes' activities on pasture and the effect of their age and gender on them. We describe and interpret a series of global and local network indices, and show that the water buffaloes differentiate their social structure in their activities on pasture and that the animals' age and gender affect their interacting patterns, and provide a framework for the application of social network analysis on grazing animals' social behavioral studies. We expect that this framework could be used in future research to provide information regarding the social structure of other kinds of animals that graze in different forage and climatic environments and help animal breeders to improve their management practices.

## 1 Introduction

Social animals living in groups develop interdependent relationships among each other, which could be either positive (affiliative) that contribute in the formation of social bonds and animals' welfare or negative (antagonistic), which could be associated with the dominance hierarchies in the animal group (Foris et al., 2019). Particularly, through affiliative interactions, such as grooming or being in close proximity and having physical contact, the animals reinforce their social bonds by expressing their preference and “friendship” towards one another (Lindberg, 2001). The succession of interactions between individuals forms their relationships whose nature, quality, and patterns result in the organization of their social structure (Hinde, 1976). The way animal societies are structured has been studied since the previous century (Whitehead, 1997). However, the application of network theory in diverse animal species, such as dolphins (Lusseau, 2003), guppies (Croft et al., 2004), chimpanzees (Watts, 2000), beef cattle (Šárová et al., 2016), sheep (Yiakoulaki et al., 2018), or bison (Ramos et al., 2019) has greatly contributed to the understanding of their social structure and the individual's social behavior and welfare.

According to the network theory, the interactions or relations among the individuals of a group can be represented as a network, where the individuals are the vertices (or nodes) and the interactions/relations among them are the edges (or ties) of the network (Coleing, 2009). Thus, in an animal population, the vertices may be individual animals or groups of animals, and the edges represent any type of social behavior, such as affiliative or antagonistic interactions (Krause et al., 2007). Social network analysis with the use of diverse indices can explicitly describe the global (at network level) and local (at vertex level) properties of a network (Sueur et al., 2011). It has only recently been applied to grazing livestock, such as sheep, to investigate the social structures that are developed among them when grazing different plant functional groups (Yiakoulaki et al., 2018).

Water buffaloes (*Bubalus bubalis*) are considered sociable rather than aggressive grazing animals (Tsiobani et al., 2016). Even though other herbivores' social structure has been studied in extent (Tucker, 2018), research concerning water buffaloes so far has focused mainly on their productive characteristics (Dezfuli et al., 2011; Bampidis et al., 2012; Singh et al., 2013), classification through their genetic origin (Kumar et al., 2007), disease transmission (Villanueva et al., 2018), welfare (Tripaldi et al., 2004; Vijayakumar et al., 2009), impact of grazing on wetlands (Georgoudis et al., 1999; Gulickx et al., 2007; Sweers et al., 2013), and grazing behavior (Napolitano et al., 2007; Antkowiak et al., 2012; Tsiobani et al., 2016). According to the latter cited authors, water buffaloes perform diverse activities during their daily routine on pastures, such as grazing, moving standing, wallowing, ruminating, lying, and drinking water. Regarding their social behavior, Napolitano et al. (2013) based on empirical observations supported that water buffaloes prefer to move in groups maintaining close proximity to one another, while particular knowledge has been provided concerning the establishment of dominance hierarchies (Madella-Oliveira et al., 2012), their antagonistic interactions (Napolitano et al., 2009), and their grooming behavior to conspecifics (De Rosa et al., 2009). However, the way water buffaloes form their social structure during their activities on pastures is still unknown. Moreover, according to our knowledge, there is no study concerning the social structure of water buffaloes with the use of network analysis methodology.

The aim of this study was to determine (a) the social structure of water buffaloes during their activities on pasture (grazing, moving, standing, ruminating, wallowing, lying, and drinking) based on their affiliative interactions (proximity), and (b) the effect of water buffaloes' age and gender on the social structure of the herd in all the abovementioned activities. We hypothesize that water buffaloes would develop different patterns of social structure in each activity on pasture. Furthermore, the age and gender of water buffaloes would influence these patterns.

## 2 Material and methods

### 2.1 Study area and experimental animal characteristics

The study was carried out at the grazing lands of Chrysochorafa (latitude 41${}^{\circ }$1046.67${}^{\prime }$ N, longitude 23${}^{\circ }$1008.48${}^{\prime }$ E), located on the southeast side of the Lake Kerkini in northern Greece, from May 2015 to April 2016. The climate of the area has Mediterranean characteristics, with hot dry summers and cold wet winters. The annual mean air temperature during the study period was 15.8 ${}^{\circ }$C, and total precipitation reached 550.2 mm (Weather Station of Chrysochorafa, 2016). The grazing lands constituted of natural grasslands, cultivated pastures, and crop residue after harvesting.

The herd that was used for the study consisted of 91 water buffaloes; 88 females (from 6 months to 23 years old; the mean age was 9 years) and three males (2.5–3 years old) that were present in the herd for reproductive purposes. No suckling calves were present in the herd; however, some adult female animals had kinship affiliations. The water buffalo herds mainly consist of adult females, a practice that is commonly used by farmers in the study area. To identify the animals, we used numbered collars with a unique number for each one of them. Two herders guided the herd to grazing lands every day and back to the stable in late afternoon. Their role was to drive the animals to places with better forage quality according to the season and also to prevent them from entering into cultivated lands. The herders kept a long distance from water buffaloes in order not to disturb their activities.

### 2.2 Measurements of animal behavior

The observations were carried out with the focal sampling technique (Altmann, 1974) during the 1-year period. An observer was following the herd for 5 h daily (from 11:00 to 16:00 LT) during 2 consecutive days at the end of each month, recording the activities of water buffaloes on pasture in 30 min time steps. The observer had spent several hours with the buffaloes before the onset of observations in order for the animals to be accustomed to their presence. The observer always started the observations from the front of the herd, covering each time the entire herd from the beginning, approaching the animals at approximately 3–4 m to identify them. The observer focused on each animal for 18 s, recording the activity that the animal performed and the conspecific(s) with whom the focal animal was in proximity. Seven activities were selected as the most representative of water buffaloes' daily routine: grazing, moving, standing, ruminating, wallowing, lying, and drinking (Tsiobani et al., 2016), which were defined as follows:

- Grazing is when the animal grazes or browses with the head down.
- Moving is when the animal moves without grazing or runs.
- Wallowing is when the animal goes into the water by either moving or standing in an upright position or lying down.
- Standing is when the animal stops every other activity and remains inactive in an upright position.
- Ruminating is when the animal either lies down or stands in an upright position while rechewing the cud.
- Lying is when the animal lies on the ground with no ruminating.
- Drinking is when the animal stands beside a watering point with the head down drinking water.

The activities were mutually exclusive, meaning that an animal could perform only one activity per observation. The proximity between two animals was recorded when the animals performed the same activity and had up to 1 m distance maximum between them. Animals that had physical contact, either with their heads or bodies, were also considered to be in proximity. Thus, one observation per animal was obtained at each 30 min time step. In total, we collected data from 240 observation periods (12 months × 2 observation days × 5 observation hours × 2 time steps per hour). The age and gender of the water buffaloes were also recorded.

### 2.3 Network and statistical analysis

#### 2.3.1 Construction of the networks

The whole water buffalo herd was considered as a network: the water buffaloes represented the vertices, and the occurrence of proximity between them formed the edges. Data were categorized according to each activity of water buffaloes on pasture and were inserted into adjacency matrices, in which the rows and columns (in identical order) represented individual animals (the vertices) and the respective cell of the section between rows and columns represented the relation (the edge) between any two animals. The matrices were binary; that is, they contained only “0” and “1” values indicating the absence and presence, respectively, of proximity between two animals in a specific activity. Also, the diagonal of the matrices was set to zero, as the animals cannot be in proximity with themselves. Therefore, as proximity was a reciprocal interaction, the matrices formed simple undirected networks. In the cases where an activity did not occur during an observation period, an empty network was formed, meaning that the matrix contained only zero values (Wasserman and Faust, 1997). Each matrix represented the network of proximities of the water buffaloes for a given activity on pasture in a specific time step.

#### 2.3.2 Indices of the networks

The networks obtained for each activity of water buffaloes on pasture were analyzed at a global and local level. The global indices, that we have used to describe basic structural characteristics of the network as a whole (Sueur et al., 2011), were density, number of components, and clustering coefficient. The local indices were the centralities of the vertices, such as the degree, closeness, betweenness, and eigenvector centrality. These indices quantify different aspects of an individual's position within the network (Makagon et al., 2012). The definition, interpretation, and mathematical formulae of all global and local indices are presented in Table 1. All network analysis was implemented with the igraph package (Csárdi and Nepusz, 2010) into the R language (R Core Team, 2017).

**Table 1:** Definition, interpretation, and mathematical formulae of the network indices.

Index		Definition – interpretation	Mathematical formula
Global	Density D	Density is the ratio of present vertices in the network to all possible edges in a network with the same number (N) of vertices (Newman, 2018). It shows how sparse or dense the network is (Wasserman and Faust, 1997).	$D=\frac{\sum_{i,j}\alpha_{ij}}{N^{2}-N}$, where $\alpha_{ij}$ with i,j=1,2,…,N are the elements of the adjacency matrix of the network (Newman, 2018).
	Number ofcomponents	The vertices and edges of any network split in a unique number of connected components. In each component, all vertices are connected via some path, and there is no path connecting the nodes of different components. The number of components shows the fragmentation of the network into mutually non-communicating parts (Harary, 1969; Newman, 2018).	The number of components is the number of irreducible components of the adjacency matrix of the network (Newman, 2018).
	Clusteringcoefficient CC	The clustering coefficient measures the degree to which the neighbors of the vertices are also connected with each other (Watts and Strogatz, 1998), the neighbor density. Networks with high clustering coefficient are made up of highly interconnected social units (Newman, 2003).	$\mathrm{CC}=\frac{1}{N}\sum_{i=1}^{N}{\mathrm{CC}}_{i}$, where CC is the clustering coefficient, and CC${}_{i}$ is the ratio of the number of links among the vertices adjacent to vertex i to the number of possible links among the vertices adjacent to i (Watts and Strogatz, 1998).
Local	Degreecentrality deg${}_{i}$	Degree is the number of direct links of the vertex i to the other vertices. The degree of the vertex i estimates the sociability of the vertex (Borgatti, 2005). Vertices with a high degree are also called hubs of the network, a term inspired by the world wide web (Newman, 2018).	deg${}_{i}=\sum_{j=1}^{N}\alpha_{ij}$, where $\alpha_{ij}$ with i,j=1,2,…,N are the elements of the adjacency matrix of the network (Newman, 2018).
	Closeness centrality Cl${}_{i}$	Closeness is a measure that describes how close a vertex i is to any other vertex j, based on both direct and indirect connections. It describes how quickly an individual can interact and communicate with others without intermediaries (Yang and Knoke, 2001).	${\mathrm{Cl}}_{i}=\frac{1}{\sum_{j=1}^{N}d_{ij}}$, where $d_{ij}$ is the number of edges in the geodesic (shortest) path linking vertices i and j (Wasserman and Faust, 1997).
	Betweenness centrality $b_{i}$	Betweenness measures the extent to which a vertex j lies on paths between other vertices i and k. Individuals with high betweenness are highly inclusive (Yiakoulaki et al., 2018) by virtue of linking together many other individuals, having thereby an influential role on the flow of resources through the network (Freeman, 1979; Lusseau and Newman, 2004; Whitehead, 2008).	$b_{i}=\sum_{i<k}\frac{g_{j(i)k}}{g_{jk}}$, where $g_{j(i)k}$ is the number of geodesic paths connecting j and k passing through i, and $g_{jk}$ is the total number of geodesic (shortest) paths connecting j and k (Borgatti et al., 2013).
	Eigenvector centrality $e_{i}$	Eigencentrality quantifies the position an individual has into a network considering how well connected that individual's direct connections are (Bonacich, 2007). Eigencentrality expresses the chain hierarchies shaped in a network by the successive contacts of the important (influential) vertices with other also important vertices (Bekiari and Hasanagas, 2015).	The eigencentrality $e_{i}$ of the vertex i is the i coordinate of the Perron–Frobenius eigenvector of the adjacency matrix ($a_{ij}$), with i,j=1,2,…,N of the network (Newman, 2018). $\left(\begin{array}{ccc} a_{11} & \cdots & a_{1N}\\ \vdots & \ddots & \vdots \\ a_{N1} & \cdots & a_{NN} \end{array}\right)\left(\begin{array}{c} e_{1}\\ \vdots \\ e_{N} \end{array}\right)=z_{\mathrm{FP}}\left(\begin{array}{c} e_{1}\\ \vdots \\ e_{N} \end{array}\right)$ The Perron–Frobenius eigenvalue ($z_{\mathrm{FP}}$) is the eigenvalue with the largest absolute value.

#### 2.3.3 Statistical procedures

Three data sets were used for the statistical analysis. The first one contained the values of global indices and the activities of water buffaloes on pasture, and the second contained the local indices also combined with the activities. In both cases, the global and the local indices were considered as dependent variables, while the activities were the independent variables. A Spearman rank correlation was implemented to estimate the association of the activities with the structural characteristics of the networks (first data set) as well as with the interacting patterns of the vertices in the network (second data set). The third data set included the non-network attributes, the age and gender of the animals (1 = female, 2 = male) along with their centralities (local indices). The association of the non-network attributes of the vertices with their centralities was also tested with the Spearman rank correlation test. All statistical procedures were carried out with the use of SPSS v.25 software (SPSS v. 25, 2017). Correlations were considered to be significant at the p levels of 0.01 and 0.05.

## 3 Results

**Figure 1:**
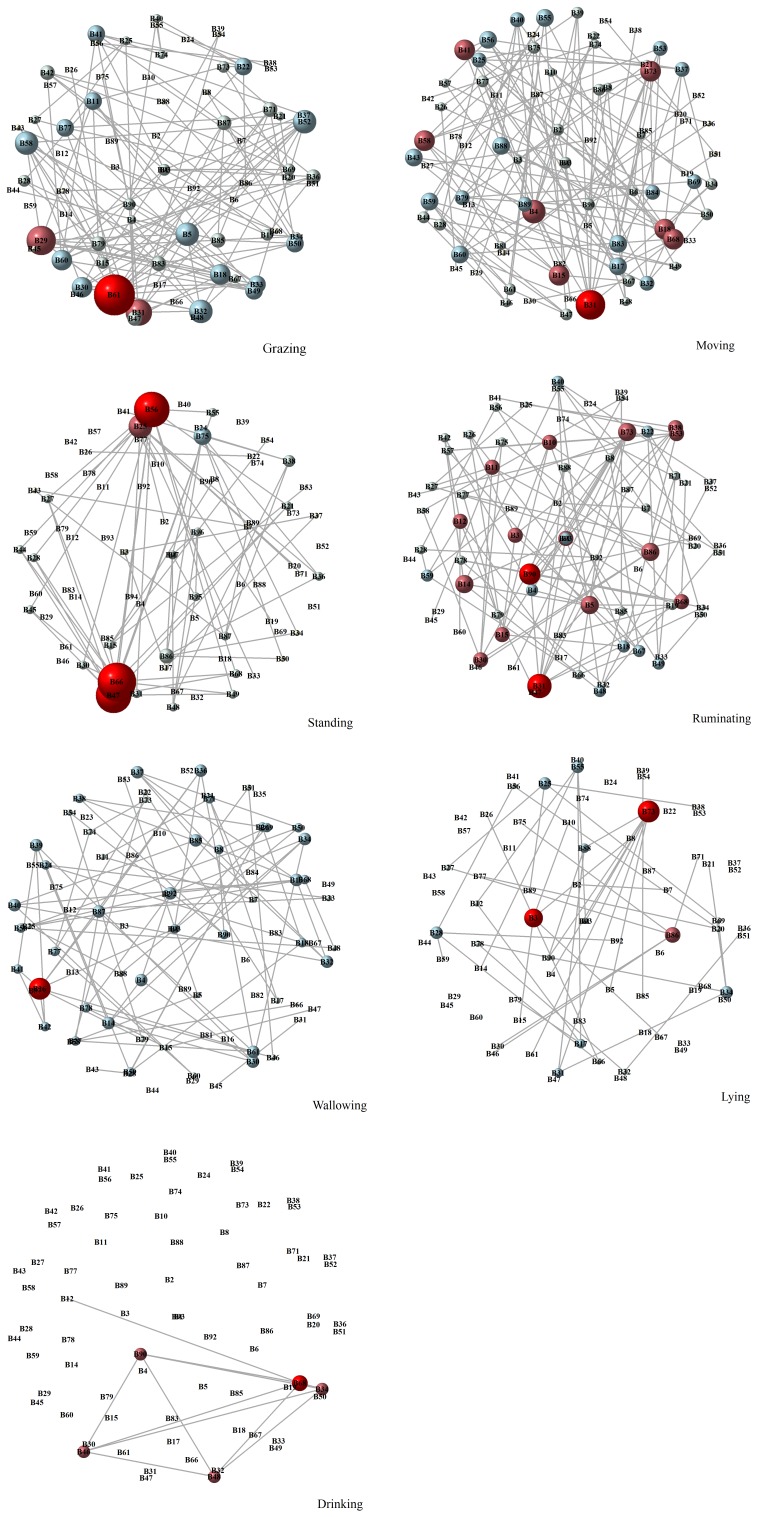
Water buffaloes' networks during their activities on pasture (grazing, moving, standing, ruminating, wallowing, lying, and drinking). The grey lines represent the edges and the circles represent the vertices of the network. An edge corresponds to the proximity between two water buffaloes. The size and color of the vertices were based on degree centrality values. Bigger and redder vertices have higher degrees, while smaller and more grey vertices have smaller degrees. The vertices' label is their identification name.

In total, we obtained 1680 simple undirected networks for all the activities of water buffaloes on pasture (7 activities × 240 observations periods) and processed 152 880 cases (1680 networks × 91 vertices). Given the high number of the obtained networks, we decided to visualize the network with the highest density from each activity to indicate the case with the most significant network effect (the added value of the interdependencies is higher when the network density is higher). Network visualization for each activity was based on the degree centrality of the vertices (Fig. 1).

### 3.1 Global indices of water buffaloes' networks

**Figure 2:**
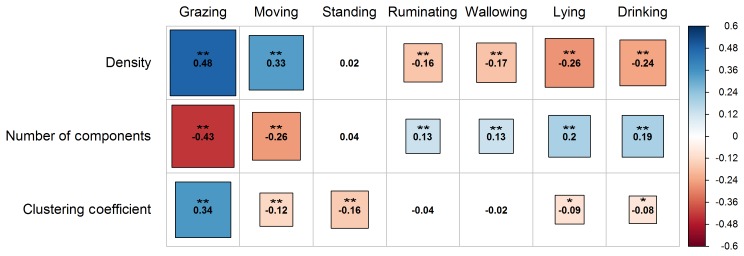
Correlogram presenting the pattern, the strength, and the significance level of Spearman's rank correlations (two-tailed) between the global indices (density, number of components, and clustering coefficient) and water buffaloes' activities on pasture (grazing, moving, standing, ruminating, wallowing, lying, and drinking). Red and blue colors represent negative and positive correlation, respectively. Darker colored and bigger square boxes in the panels correspond to higher strength of correlation. Asterisks indicate the statistically significant level of association at P<0.05 (${}^{*}$) and P<0.01 (${}^{**}$).

The Spearman correlation test revealed that the indices of density, number of components, and clustering coefficient were significantly correlated with the activities of water buffaloes on pasture (P<0.01 and P<0.05) (Fig. 2). Particularly, density was positively associated with grazing and moving (P<0.01), though the association of grazing was stronger compared to moving. Also, density was insignificantly associated with the activity of standing (P>0.05) and negatively correlated with the activities of ruminating, wallowing, lying, and drinking (P<0.01). In addition, the number of components had a negative association with the activities of grazing and moving (P<0.01), though moving seemed to have a less strong negative correlation than grazing. Also, this index was not related to the activity of standing (P>0.05), and it was positively associated with the rest of the activities (ruminating, wallowing, lying, and drinking; P<0.01 in all cases). With regards to the clustering coefficient, it was only positively associated with the activity of grazing (P<0.01). On the contrary, the clustering coefficient was negatively associated with the activities of moving (P<0.01), standing (P<0.01), lying (P<0.05), and drinking (P<0.05), while it was insignificantly correlated with ruminating and wallowing (P>0.05 in both cases).

### 3.2 Local indices of water buffaloes' networks

**Figure 3:**
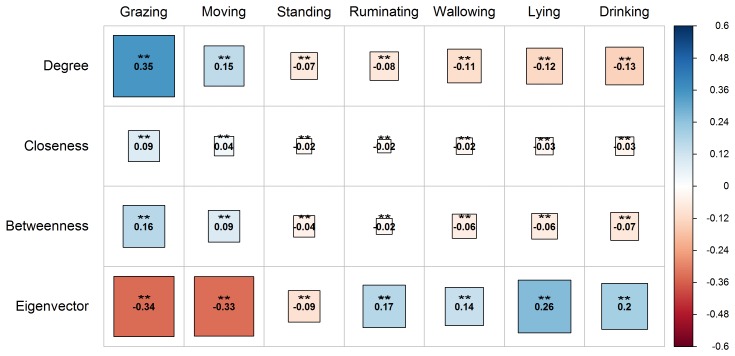
Correlogram presenting the pattern, the strength, and the significance level of Spearman's rank correlations (two-tailed) between the local indices (degree, closeness, betweenness, and eigenvector) and water buffaloes' activities on pasture (grazing, moving, standing, ruminating, wallowing, lying, and drinking). Red and blue colors represent negative and positive correlation, respectively. Darker colored and bigger square boxes in the panels correspond to higher strength of correlation. Asterisks (${}^{**}$) indicate the statistically significant level of association at P<0.01.

The local indices (centralities) of the vertices proved to be highly correlated (P<0.01) with the activities of buffaloes on pasture (Fig. 3). Particularly, the indices of degree, closeness, and betweenness centrality were positively associated with grazing and moving (P<0.01). However, the eigenvector centrality was negatively associated with the above activities (P<0.01). It is significant to highlight that grazing was strongly correlated with the degree, closeness, and betweenness centralities of the vertices compared to moving. All the centralities were negatively correlated with the activity of standing (P<0.01). During the activities of ruminating, wallowing, lying, and drinking, an opposite pattern to that of grazing and moving was observed. Particularly, the degree, closeness, and betweenness centralities had a negative association (P<0.01) with these activities, while there was a positive association with the eigenvector centrality (P<0.01).

### 3.3 Correlation of age and gender with the local indices

**Figure 4:**
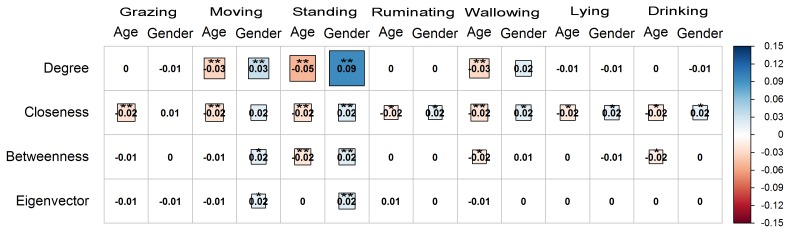
Correlogram presenting the pattern, the strength, and the significance level of Spearman's rank correlations (two-tailed) between the centralities (degree, closeness, betweenness, and eigenvector) and the attributes age and gender (female = 1, male = 2) of water buffaloes during their activities on pasture (grazing, moving, standing, ruminating, wallowing, lying, and drinking). Red and blue colors represent negative and positive correlation, respectively. Darker colored and bigger square boxes in the panels correspond to higher strength of correlation. Asterisks indicate the statistically significant correlation at P<0.05 (${}^{*}$) and P<0.01 (${}^{**}$).

The age of the water buffaloes was negatively correlated with the degree centrality in the activities of moving, standing, and wallowing (P<0.01), while it was not correlated (P>0.05) with the activities of grazing, ruminating, lying, and drinking (Fig. 4). Also, the age of the animals was negatively correlated with the closeness centrality in all the observed activities on pasture (P<0.01 for grazing, moving, standing, and wallowing; P<0.05 for ruminating, lying, and drinking). It was also negatively associated with the betweenness centrality in the activity of standing (P<0.01), wallowing (P<0.05), and drinking (P<0.05), while it was not correlated with the rest of the activities (P>0.05). On the other hand, the eigenvector centrality had an insignificant correlation with age in all the activities of water buffaloes on pasture (P>0.05).

The gender of the water buffaloes was not correlated with any centrality (P>0.05) in the activity of grazing. It was positively associated with the degree in moving and standing (P<0.01), while it was not correlated with the activities of ruminating, wallowing, lying, and drinking (P>0.05). The gender was positively associated with the closeness centrality in the activities of standing (P<0.01), ruminating, wallowing, lying, and drinking (P<0.05), while the correlation with moving was not statistically significant (P>0.05). Also, the attribute of gender was positively correlated with betweenness and eigenvector centrality in the activities of moving (P<0.05) and standing (P<0.01), while no correlation was observed with the rest of the activities (P>0.05) for these indices.

## 4 Discussion

Water buffaloes during their activities on pasture seem to clearly differentiate their social structure as well as their interacting patterns. Specifically, during grazing and moving, water buffaloes tended to form dense networks, while the opposite was observed in the activities of ruminating, wallowing, lying, and drinking. During grazing, the animals kept their cohesion (positive correlation with density) and their compactness (negative correlation with the number of components) developing simultaneously strong social bonds with their partners (positive association with the clustering coefficient). These bonds offer fitness benefits in terms of animals' survival or reproductive success (Firth et al., 2015). Our results are consistent with Bouissou (1980), who reported that free-ranging cattle living in natural environments enjoy greater cohesion and less antagonism than cattle in intensive breeding systems due to the limited competition for food resources and the ample personal space. During grazing, water buffaloes increased their contacts, as indicated by the degree (direct contacts) and closeness centrality (indirect contacts), demonstrating high levels of sociability. This is understandable, as water buffaloes are gregarious animals, and not only do they freely express their natural behavior during grazing but they also interact with their conspecifics (Napolitano et al., 2013). The strong social cohesion of water buffaloes was intensified by their tendency to form large groups including most of their partners (positive association with the betweenness centrality) and consequently minimizing the isolation. In this regard, Jensen (2018) stated that cattle perceive isolation as an aversive characteristic and that they demonstrate signs of increased stress when isolated or deprived of their herd partners (Raussi et al., 2003; Færevik et al., 2006). At a deeper level, the grouping and the inclusive tendency of water buffaloes might result in the formation of small subgroups like “neighborhoods”. This structure was observed only during the activity of grazing. This finding is consistent with Sowell et al. (2000) and Ungerfeld et al. (2014), who reported that during grazing cattle form subgroups through which they learn and develop different foraging strategies in relation to the behavior of the other members of the herd. Additionally, the lack of hierarchies as depicted through the eigenvector centrality could be attributed to the fact that the socializing implied above does not allow the appearance of acute ranking differences. In general, the process of grazing is considered a more welfare-friendly system for animals (Koidou et al., 2019), and hence the social character of grazing could lead to better communication among water buffaloes, resulting in the improvement of their welfare and their ability to implement optimal foraging strategies. Furthermore, during the foraging process, the animals' opportunities for establishing an important position in the herd seemed to be independent of age and gender. However, as revealed by the negative association of age with closeness centrality, elder water buffaloes tended to avoid interaction with their conspecifics during the activity of grazing.

The activity of moving also proved to be a social activity like grazing, as indicated by the density, the degree, closeness, and betweenness centrality. Similarly, the necessity of maintaining social coherence under conditions of mobility in grazing cattle was also stated by Sato (1982). However, due to the fact that moving is an antagonistic activity, water buffaloes did not tend to socialize as much as during grazing. Also, water buffaloes did not tend to form groups during moving, as indicated by the negative association of components with this activity. In this regard, Moran and Doyle (2015) stated that during moving cattle form a compact whole and move all together, as the movement of one animal urges the other ones to move too. However, according to our results, water buffaloes constituted a more dispersed network (negative association with the clustering coefficient) with no hierarchical tendencies (negative association with the eigenvector). According to Moran and Doyle (2015), cattle usually prefer to keep order during moving, without overtaking each other. However, possible external disturbances (e.g., influence of the guardians) might have a structural effect during the animals' movement. Nevertheless, this was not the case in our research, because the guardians kept a long distance from the herd during our observations, as stated in the material and methods section. Moreover, the elder animals seemed to be more isolated from their conspecifics during moving, considering that they do not have direct and indirect relations (negative association of degree and closeness centralities with age). This is probably attributed to the inability of aged animals to keep up with the pace of the herd (Manning and Dawkins, 2012). On the other hand, the male individuals seemed to gain both in sociality and hierarchy during this activity; given that they developed more relations (direct or indirect), they were more inclusive with their partners and built chain hierarchies.

The activity of standing seemed to be inappropriate for socializing, as the animals displayed individualization tendencies (negative correlation with the clustering coefficient and all the centralities as well). However, during this activity, male buffaloes seemed to be more sociable, inclusive, and more important in terms of the herd's social ranking, as indicated by the positive association of this activity with all four centralities. This could be the case particularly during the reproduction period when females seek the males and stand still accepting the male contact. Thus, the estrus-related attractiveness of male buffaloes is strengthened due to their small number and their scarcity within such a large herd of female animals, resulting even in the enhancement of their leading potential (positive association with the eigenvector centrality). The isolation and the degradation of aged individuals were also obvious in terms of direct contacts, lack of importance, and inclusiveness (negative correlation of age with the degree, closeness, and betweenness centralities), as well as the deconstruction of hierarchies. Similarly to our results, Ramos et al. (2019) stated that there was no social association preference of a similar age bisons or individuals' dominance rank within the herd.

The coherence of the herd tended to decrease during the activities of ruminating, wallowing, lying, and drinking. The negative association of these activities with the density index as well as with degree, closeness, and betweenness centralities indicated that the water buffaloes seemed to be less sociable when performing the above activities. However, their positive association with the eigenvector centrality index revealed that water buffaloes tended to develop proximity relations with the most important individuals building chain hierarchies. This is more evident in the activity of lying, followed by the activities of drinking, ruminating, and wallowing. Particularly, in the activity of lying, water buffaloes tended to decrease their contacts, direct and indirect ones, and form more sparse networks that were characterized according to our observations by many isolated individuals and distinct subgroups. This is understandable, as water buffaloes on the open area of grasslands have sufficient and comfortable lying space in contrast to confined conditions. On the other hand, lying is not a frequent activity of water buffaloes, as they spend only 0.94 % of their time on it (Tsiobani et al., 2016). Water buffaloes spent more time on grazing and moving on grasslands depending on the amount and the quality of the available forage. Hence, they devote less time to lying during grazing, which has a negative effect on the resting aspect of their welfare.

Regarding ruminating, the buffaloes also present a tendency to deconstruct individual proximities and to increase the formation of subgroups. This finding is in accordance with the results of Grant and Albright (2006). Additionally, the formation of chain hierarchies during this activity is in accordance with Ungerfeld et al. (2014), who reported that the hierarchies formed in a cattle herd affect the activity of rumination. Particularly, they stated that animals of lower social ranking ruminated 35 % less than animals in higher social ranking. When cattle ruminate, they prefer to lie down, lower their head, and close their eyes (Grant and Dann, 2015). This state of relaxation could resemble the state of drowsiness (Karasabbidis et al., 2014). Rumination seems to occur as intermittent to their daily foraging routine and significantly depends on the quality of the forage consumed (Wang et al., 2018). Moreover, according to the cited authors, the actual proportion of time spent ruminating is affected by the age and sex of an individual.

As for the activity of wallowing, water buffaloes displayed a trend similar to what was described for ruminating. The activity of wallowing helps water buffaloes to remove excess body heat and also protects them against biting flies and other ectoparasites (Hafez and Shafei, 1954). Water buffaloes do not strengthen their sociability within the herd but they rather prefer to connect with the most important animals forming distinct subgroups, as implied by the positive association with the components and the eigenvector.

Concerning drinking, a more isolative tendency of water buffaloes from the other members of the herd was observed. However, chain hierarchies were also reinforced, as indicated by the positive association of this activity with the eigenvector centrality index. The activity of drinking is an activity of vital importance, directly related to the survival, performance, and welfare of water buffaloes. In this regard, Schein and Fohrman (1955) have observed that when a cow stops grazing and starts to walk towards the watering point, often another cow starts following her in the specific activity.

The age of animals did not seem to constrain water buffaloes from developing proximity relations during ruminating, lying, and drinking. However, during wallowing, the negative association of age with the degree, closeness, and betweenness centrality implies the isolation and degradation of aged individuals. In this regard, Bøe and Færevik (2003) reported that young animals have higher rates of social interactions than adult ones. Nevertheless, in female herds, as reported by Reinhardt et al. (1986), social ranking is related to the age of the animals. In our study, even though the herd consisted mainly of females, hierarchies related to the age of the animals were not detected. However, during ruminating, wallowing, lying, and drinking, male animals seemed to be more sociable, developing either direct or indirect relationships.

## 5 Conclusions

In conclusion, we found that the sociability and compactness of the herd were enhanced during grazing and moving, with no acute hierarchies. However, water buffaloes revealed a cliquish structure through the formation of small “neighborhoods” only in the activity of grazing. During ruminating, wallowing, lying, and drinking, the buffaloes seemed to be less sociable, although they tended to reinforce hierarchies, which was more evident in the activity of lying. Additionally, during ruminating, they tended to increase the formation of subgroups, while during drinking they developed more isolative tendencies, forming chain hierarchies. Elder individuals seemed to be isolated during grazing and moving, and they showed tendencies of degradation in standing. During ruminating, lying, and drinking, they developed proximity relations with the most important individuals. Male buffaloes gained in sociality, inclusiveness, and importance in herd's social ranking during moving and standing.

Our data shed light on the understanding of social links and interactions that occur among water buffalo individuals during their activities on pasture as well as in the structure of the whole herd. Extending the methodological approach of social network analysis to other kinds of animals that graze in different forage environments and climatic conditions will be an interesting topic for future studies. Such information would contribute to a more conclusive understanding of grazing animals' social behavior and organization and help improve their management practices and welfare.

## Data Availability

The original data are available upon request to the corresponding author.
